# Associations between trait mindfulness and physical activity: the parallel mediating effect of exercise positive well-being and exercise psychological distress

**DOI:** 10.1186/s40359-025-02751-8

**Published:** 2025-04-22

**Authors:** Zijian Zhao, Youteng Gan

**Affiliations:** 1 School of Physical Education, Guangdong University of Education, Guangzhou, 510800 China; 2 School of Psychology, Beijing Sport University, Xinxi Road 48, Haidian District, Beijing, 100084 China

**Keywords:** Trait mindfulness, Physical activity, Exercise affect, Longitudinal tracking

## Abstract

**Objective:**

An increasing body of evidence suggests that exercise positive affect contribute to sustained engagement in physical activity, yet few studies have proposed intervention strategies targeting exercise affect. Currently, mindfulness is considered to have the potential to promote the improvement of exercise affect and physical activity. This study combines cross-sectional design and longitudinal design to explore the relationship between trait mindfulness and physical activity, and examines whether exercise positive well-being and exercise psychological distress mediate this relationship.

**Methods:**

At first, this study adopted a large sample cross-sectional design, and assessed the participants’ trait mindfulness, exercise positive well-being, exercise psychological distress and physical activity through online self-report survey (*n* = 853, male = 54.7%; M_age_ = 19.16, SD = 0.97). Subsequently, a subset of participants from the cross-sectional study (*n* = 354, male = 52.8%; M_age_ = 19.21, SD = 0.86) were followed up for three months by longitudinal follow-up design, and the time relationships between these variables was examined.

**Results:**

In the cross-sectional survey, the results of the correlation analysis showed that trait mindfulness, exercise positive well-being, and physical activity were all significantly positively correlated, while psychological distress was negatively correlated with the other variables. Similar correlations were observed during longitudinal follow-up. Both cross-sectional and longitudinal mediation analyses indicated that exercise positive well-being and exercise psychological distress served as parallel mediators in the relationship between trait mindfulness and physical activity.

**Conclusion:**

Exercise positive well-being and exercise psychological distress may serve as potential mechanisms through which trait mindfulness influences physical activity. This provides a theoretical basis and practical direction for future development of mindfulness-based interventions to promote physical activity.

## Introduction

Physical activity is defined as any bodily movement produced by skeletal muscles that results in energy expenditure [1]. It is widely recognized for its positive effects on physical health [2, 3]. Research has demonstrated that physical activity can effectively improve sleep quality [4], alleviate obesity [5, 6], reduce depression [7], and mitigate risks associated with cardiovascular diseases [8] and metabolic syndrome [9], among other chronic conditions. However, approximately 27.5% of adults worldwide are estimated to be insufficiently physical active [10], with an alarming 81% of adolescents failing to meet recommended physical activity levels [11]. Even more concerning, physical inactivity has been identified as the fourth leading risk factor for global non-communicable disease mortality [12].To address this issue, numerous researchers and organizations have been actively reporting on and advocating for increased physical activity from a global perspective, aiming to encourage more individuals to engage in regular physical activity [13–16].

How can we encourage people to start exercising and spend more time engaging in physical activity? Researchers have explored the relationship between cognitive mediators and physical activity from the perspective of rational decision-making, such as beliefs about consequences, perceptions of capability, perceived social influences, and self-regulation. However, the actual effects of these cognitive mediators, which are considered to be beneficial to initiate or sustain exercise, have not been particularly promising [17, 18]. Indeed, an increasing number of researchers have begun to focus on the affective mechanisms underlying exercise or physical activity. According to Russell (2003), affect is conceptualized as “a neurophysiological state consciously accessible as the simplest raw (nonreflective) feelings”, and it plays an important role in regulating human behavior and influences behavioral reflexes, motivational processes underlying various behaviors as well as complex decision making [19]. They argue that exercise positive affect can promote exercise behavior [20, 21], while exercise negative affect can hinder more frequent participation in physical activity [21]. Exercise affective experiences are considered a crucial factor influencing both current exercise behavior and future exercise motivation. Therefore, finding effective methods to enhance exercise positive affect is of great significance for promoting physical activity.

In recent years, mindfulness has gained increasing attention due to its significant potential in health behavior interventions, particularly in promoting physical activity [22, 23]. Mindfulness refers to paying attention to what is happening now with a non-judgmental attitude [24], and mindfulness interventions were first applied in Mindfulness-Based Stress Reduction (MBSR), which includes an 8-week mindfulness program, with practices such as body scan and breath awareness. It emphasizes focusing on the present moment and gently redirecting attention back to the present when it wanders [25]. And mindfulness has also been found to have a significant positive correlation with physical activity [26], making it an important factor influencing physical activity. For example, Sala et al. (2021) conducted a one-week mindfulness intervention for adults with insufficient physical activity and found that the mindfulness group significantly increased the time spent in moderate-to-vigorous physical activity compared to the control group [23]. A meta-analysis by Schneider et al. (2019) also found that mindfulness-based interventions had a positive impact on participants’ physical activity, with a positive correlation between mindfulness and physical activity, particularly psychological factors related to physical activity [27].

In addition, mindfulness has also been shown to be effective in promoting positive affective experiences [28]. Mindfulness is considered an effective affective regulation strategy, as its two core components: attention to the present moment and a non-judgmental attitude, can increase positive affective experiences and reduce negative affective experiences [29]. The mindful emotion regulation model introduces a possible connection between mindfulness and emotion regulation. Chambers et al. (2009) suggest that mindfulness-based emotion regulation should enhance systematic training in “awareness” and “nonreactivity,” rather than suppressing, reevaluating, or changing affective experiences in any way. Through mindfulness, individuals consciously choose the thoughts, emotions, and sensations they will identify with, but without engaging in habitual reactions, thus gradually eliminating the automatic evaluation process of negative affect [30].

The relationship between mindfulness and both positive and negative affective experiences has been supported by numerous studies. For example, Mandal et al. (2012) recruited 100 students and had them self-report on mindfulness, positive affective experiences, and negative affective experiences [31]. The results showed that mindfulness scores were positively correlated with positive affective experiences and negatively correlated with negative affective experiences. Trait mindfulness, a subconcept of mindfulness, refers to an individual’s characteristic tendency to maintain awareness of the present moment in a nonreactive and nonjudgmental way [32], and is believed to remain relatively stable over time [24]. Jose & Geiserman (2024) explored the longitudinal relationship between trait mindfulness and affect using a random intercept cross-lagged model, finding that trait mindfulness could predict participants’ negative affective experiences, which decreased over time [33]. Additionally, researchers using the subjective exercise experience scales to assess individuals’ affective experiences during exercise found a significant correlation between trait mindfulness and exercise affective experiences. Individuals with higher trait mindfulness levels demonstrated significantly more positive exercise experiences [34]. Ticklay & Jones (2024) examined the impact of listening to mindfulness recordings during outdoor low-to-moderate intensity walking on exercise affective experiences, finding that, compared to the control group, the mindfulness group experienced more positive affective responses during and after the walk [35]. The findings of these studies highlight the close relationship between mindfulness and positive affective experiences and demonstrate its positive impact on exercise affective experiences.

Regarding the relationship between exercise affect and physical activity, Ekkekakis (2003) proposed the Dual-Mode Theory (DMT), which emphasizes the role of affective responses in exercise behavior [36]. According to this theory, affective responses in exercise are influenced by cognitive factors and interoceptive stimuli with different intensities, with the relative influence of these factors changing based on exercise intensity [37]. Moreover, Williams & Evans (2014) put forward the Affect and Health Behavior Framework (AHBF), which categorizes affective responses to health behaviors into immediate affective responses and post-behavioral affective responses [38]. In terms of physical activity, they suggested that positive and negative affective experiences from previous exercise behaviors can either increase or decrease the likelihood of future exercise engagement. Liao et al. (2017) explored the connection between affective experiences during previous physical activity and future physical activity [39]. The study found that an increase in exercise positive affective experiences or a decrease in exercise negative affective experiences could predict an increase in future physical activity. The Affective-Reflective Theory (ART) of physical inactivity and exercise, a recently recognized dual-process model, suggests that exercise-related stimuli trigger automatic associations, leading to an affective valuation of exercise (type-1 process). This valuation then informs a reflective evaluation (type-2 process), provided self-control resources are available. The automatic affective valuation, which assigns positive or negative affective valence to exercise-related stimuli, plays a key role in initiating physical activity [40].

Recently, Stevens et al. (2020) organized the Affect and Health Behavior Framework to provide a clearer definition and distinction of the various affective structures related to physical activity. These affective structures are categorized into: affective response, incidental affect, affect processing, and affectively charged motivational states [41]. They presented specific examples of how the framework can be applied in exercise behavior interventions, emphasizing that affective factors are central in determining physical activity motivation. In summary, based on the Affect and Health Behavior Framework, exercise affective experiences are regarded as an important factor influencing physical activity.

Stevens et al. (2020) suggested that considering affective responses to physical activity as a mediator could be beneficial for designing more targeted physical activity interventions [41]. However, there has been limited research exploring the role of exercise positive affective experiences and exercise negative affective experiences in the relationship between trait mindfulness and physical activity. Therefore, based on the Affect and Health Behavior Framework, our study aims to investigate the relationship between trait mindfulness and physical activity, specifically exploring whether exercise affective experiences could serve as a mediator between the two. This will help us better understand the mechanisms through which trait mindfulness promotes physical activity. In this study, we conducted a cross-sectional survey to explore the relationships among trait mindfulness, exercise positive well-being, exercise psychological distress, and physical activity. Additionally, we included several demographic variables to control for potential confounding factors in subsequent analyses. Given that cross-sectional studies cannot further explore the causal relationships between variables, we followed up with a longitudinal survey, reassessing a subset of participants three months later to examine the relationships between the four variables across time. Based on previous research findings, we hypothesize that: (1) Trait mindfulness will positively predict physical activity and exercise positive well-being, and negatively predict exercise psychological distress, while exercise positive well-being will positively predict physical activity, and exercise psychological distress will negatively predict physical activity; (2) Exercise positive well-being and exercise psychological distress will act as parallel mediators in the relationship between trait mindfulness and physical activity.

## Method

### Participants

Participants in this study were primarily recruited from several universities in southern and central China. In the cross-sectional survey, a total of 881 participants completed baseline measurements. However, 28 participants were excluded due to missing personal information or because they were under 18 years old. Ultimately, data from 853 participants (467 male, 54.7%; 386 female, 45.3%) were included in the study, with a valid response rate of 96.82%. The average age of these participants was 19.16 years (SD = 0.97), and all were enrolled in full-time associate or undergraduate education. A small number of participants (*n* = 78, 9.1%) reported having previously participated in mindfulness training.

In the longitudinal survey, due to resource limitations related to collaborators, we were unable to invite all participants from the cross-sectional survey. Therefore, we used a random sampling method to select a subset of participants for the longitudinal follow-up. A total of 367 participants voluntarily took part in the follow-up survey. Thirteen were excluded for not completing the longitudinal follow-up, leaving data from 354 participants (187 male, 52.8%; 167 female, 47.2%) who completed both the initial and follow-up surveys. The valid response rate for the follow-up survey was 96.45%. The average age of these participants was 19.21 years (SD = 0.86), and 49 participants reported having participated in mindfulness training.

In this study, two rounds of data collection were conducted: the first round is referred to as T1, and the second round as T2. The time interval between the two data collections was approximately 3 months.

### Procedure

In this study, we collaborated with researchers from several universities in different regions of mainland China to recruit student participants. These participants completed an online self-report questionnaire regarding the study. The questionnaire included demographic information, trait mindfulness, exercise positive well-being, exercise psychological distress, and physical activity. Before the survey began, we obtained informed consent from all participants and clearly explained the confidentiality and purposes of the study. Additionally, participants were informed that there were no right or wrong answers to the survey and that they had the right to withdraw from the study at any time. Furthermore, they were given the option to receive their personal survey report via email after completing the survey. This study was conducted in accordance with the principles of the Declaration of Helsinki and received approval from the local ethics committee.

### Measures

Demographic Information: This section includes age, height, weight, gender, and whether participants had prior experience with mindfulness training. Height and weight were used to calculate the body mass index (BMI).

#### Trait mindfulness

Trait mindfulness was measured using the 15-item Chinese Short Form of the Five Facet Mindfulness Questionnaire (FFMQ-C-SF) [42, 43]. The FFMQ-C-SF includes five dimensions: Observing, Describing, Acting with Awareness, Non-judging, and Non-reacting. The scale uses a 5-point Likert scale (1 = strongly disagree, 5 = strongly agree), where higher scores indicate higher levels of trait mindfulness. The FFMQ-C-SF has demonstrated good validity and reliability in Chinese populations [42]. In the current study, the internal consistency coefficient was 0.83 at T1 and 0.86 at T2.

#### The subjective exercise experience scale

The Subjective Exercise Experience Scale (SEES) was developed by McAuley and Courneya (1994) and includes three dimensions: Positive Well-Being, Psychological Distress, and Fatigue [44]. In this study, we selected only the affective-related dimensions from the scale, namely Positive Well-Being (PWB; e.g., “Participating in physical exercise makes me feel great”) and Psychological Distress (PD; e.g., “Participating in physical exercise makes me feel awful”), along with their corresponding items. Each dimension consists of four items, for a total of eight items. In the Subjective Exercise Experience Scale, the positive well-being scale is considered to measure exercise positive affective experiences, while the psychological distress scale measures exercise negative affective experiences [44]. The Subjective Exercise Experience Scale uses a 7-point Likert scale, where higher scores on the positive well-being scale indicate stronger exercise positive affective experiences, and higher scores on the psychological distress scale indicate stronger exercise negative affective experiences. The reliability and validity of the Subjective Exercise Experience Scale have been confirmed in Chinese populations [45]. In this study, the internal consistency coefficient for the positive well-being scale was 0.91 at T1 and 0.90 at T2, while the internal consistency coefficient for the psychological distress scale was 0.94 at both T1 and T2.

#### Physical activity

In this study, we used the International Physical Activity Questionnaire Short Form (IPAQ-SF) [46] to assess participants’ frequency (days per week) and total duration (minutes per day) of various physical activities (including vigorous, moderate, and walking) over the past week. The IPAQ-SF consists of seven items, six of which relate to individual physical activity levels. Total physical activity volume is calculated by summing the durations of activities across different intensities. The IPAQ-SF has shown good reliability and validity in Chinese populations [47].

### Data analysis

Based on the data from the cross-sectional survey and longitudinal follow-up survey, we conducted preliminary analysis and mediation analysis using SPSS 26.0 and the PROCESS 4.1 macro for SPSS. The preliminary analysis included descriptive statistics and examination of correlations between demographic variables and these study variables. For the mediation analysis, we used a bootstrap method with 5,000 resamples to obtain the 95% bias-corrected confidence intervals for the indirect effects [48]. A mediation effect was considered significant when the 95% bias-corrected bootstrap confidence interval did not contain zero [49].

## Results

### Cross-sectional survey results

#### Correlation analysis

After the preliminary processing of the data collected from the cross-sectional survey, we conducted descriptive statistics and correlation analysis for trait mindfulness, exercise positive well-being, exercise psychological distress, and physical activity. Since none of the variables followed a normal distribution (Shapiro-Wilk test, *p* < 0.05), Spearman’s rank correlation analysis was used in this study. The results revealed significant correlations among these four variables (correlation range: -0.359 to 0.443, *p* < 0.01). Trait mindfulness, exercise positive well-being, and physical activity were positively correlated with each other, while exercise psychological distress showed negative correlations with the other three variables, as detailed in Table 1. Additionally, we found low-level correlations between age, BMI and some of the study variables. Given that previous studies on physical activity have controlled for BMI as a covariate [50], and taking into account gender differences, we controlled for these three demographic variables(i.e., age, BMI and gender) in subsequent mediation analyses.

**Table 1:** Correlations between trait mindfulness, exercise positive well-being, exercise psychological distress, physical activity and demographic variables (*n* = 853)

	FFMQ	IPAQ	PWB	PD
FFMQ	-			
IPAQ	0.302^**^	-		
PWB	0.372^**^	0.443^**^	-	
PD	-0.279^**^	-0.357^**^	-0.359^**^	-
Age	0.086^*^	0.290^**^	0.180^**^	-0.085^*^
BMI	0.005	0.051	0.098^**^	-0.020
M ± SD	48.76 ± 6.11	1309.92 ± 2012.47	19.41 ± 5.17	12.57 ± 6.17

Note: * indicates *p* < 0.05, ** indicates *p* < 0.01; # FFMQ = Chinese version of the Five Facet Mindfulness Questionnaire-Short Form; IPAQ = International Physical Activity Questionnaire Short Form; PWB = Positive Well-Being of Subjective Exercise Experience Scale; PD = Psychology Distress of Subjective Exercise Experience Scale

#### Mediation analysis

To explore the potential relationships between the variables, we conducted a mediation analysis with trait mindfulness as the independent variable, exercise positive well-being and exercise psychological distress as mediators, and physical activity as the dependent variable. After controlling for age, gender, and BMI, the analysis was performed using the PROCESS 4.1 macro for SPSS.

**Fig. 1:**
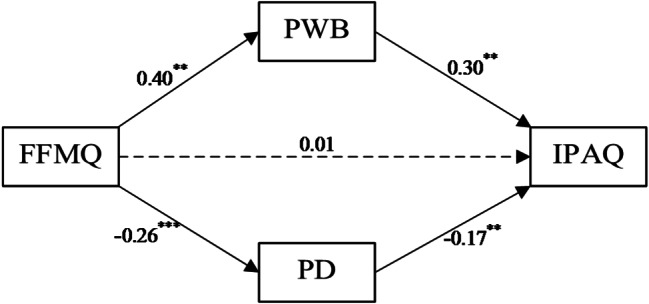
Mediating paths between mindfulness and physical activity. Note ** indicates *p* < 0.01, *** indicates *p* < 0.001; # FFMQ = Chinese version of the Five Facet Mindfulness Questionnaire-Short Form; IPAQ = International Physical Activity Questionnaire Short Form; PWB = Positive Well-Being of Subjective Exercise Experience Scale; PD = Psychology Distress of Subjective Exercise Experience Scale

As shown in Fig. 1, trait mindfulness was found to positively predict exercise positive well-being (β = 0.40, *p* < 0.01) and negatively predict exercise psychological distress (β = -0.26, *p* < 0.01). Exercise positive well-being positively predicted physical activity (β = 0.30, *p* < 0.01), while exercise psychological distress negatively predicted physical activity (β = -0.17, *p* < 0.01). These results partially support Hypothesis 1.

Based on the mediation analysis, the results showed that the total effect was 56.857, with a 95% confidence interval (CI) of [35.647, 78.066], and the indirect total effect was 54.317, with a 95% CI of [40.232, 70.600], accounting for 95.53% of the total effect. Specifically, within the indirect effects:

(1) The mediation effect for the path “trait mindfulness → exercise positive well-being → physical activity” was 39.328, with a 95% CI of [27.709, 52.963], accounting for 69.17% of the total effect.

(2) The mediation effect for the path “trait mindfulness → exercise psychological distress → physical activity” was 14.990, with a 95% CI of [8.295, 23.078], accounting for 26.36% of the total effect.

These results indicate that trait mindfulness indirectly influencing physical activity through both exercise positive well-being and exercise psychological distress as mediators. The direct effect was 2.540, with a 95% CI of [-19.981, 25.060], accounting for 4.47% of the total effect. As the confidence interval includes 0, this suggests that the direct effect is not significant. Details are provided in Table 2.

**Table 2:** Results of mediation analyses (*n* = 853)

			95% CI		
Effect Type	Estimate	S.E.	Lower 2.5%	Upper 2.5%	|IE/DE|
Total Effect	56.857	10.806	35.647	78.066	
Indirect Total Effect	54.317	7.793	40.232	70.600	95.53%
FFMQ→PWB→IPAQ	39.328	6.395	27.709	52.963	69.17%
FFMQ→PD→IPAQ	14.990	3.725	8.295	23.078	26.36%
Direct Effect	2.540	11.474	-19.981	25.060	4.47%

Note: S E = standard error, CI = confidence interval; IE = indirect effect (each path); DE = direct effect. FFMQ = Chinese version of the Five Facet Mindfulness Questionnaire-Short Form; IPAQ = International Physical Activity Questionnaire Short Form; PWB = Positive Well-Being of Subjective Exercise Experience Scale; PD = Psychology Distress of Subjective Exercise Experience Scale

### Longitudinal follow-up results

Although the hypotheses in this study were confirmed in the cross-sectional survey, a longitudinal follow-up was conducted to further explore the longitudinal relationships between the variables. Based on the cross-sectional survey, we conducted a 3-month follow-up survey with some participants. The data collected in the first survey (from the cross-sectional survey phase) were defined as T1, and the data collected in the second survey were defined as T2.

#### Correlation analysis

We conducted descriptive statistics and correlation analysis on the data from T1 and T2 for the various study variables, with the results shown in Table 3. Since none of the variables followed a normal distribution (Shapiro-Wilk test, *p* < 0.05), Spearman’s rank correlation analysis was used in this study. The results indicated that the four variables at T1 and T2 were mostly significantly correlated to varying degrees (correlation range: -0.430 to 0.589, *p* < 0.05), with the exception of IPAQ (T1) and FFMQ (T2), which did not exhibit a significant correlation. These correlations support the consistency and stability of the variables across the two time points (T1 and T2), and suggest that the relationships observed in the cross-sectional data can be observed over time as well.

**Table 3:** Correlations between trait mindfulness, exercise positive well-being, exercise psychological distress and physical activity(*n* = 354)

	T2	FFMQ	IPAQ	PWB	PD
T1	M ± SD	50.15 ± 6.06	580.42 ± 449.74	21.04 ± 4.44	12.57 ± 6.34
FFMQ	50.29 ± 6.00	0.578^**^	0.109^*^	0.307^**^	-0.309^**^
IPAQ	2084.52 ± 2286.33	0.035	0.589^**^	0.276^**^	-0.147^**^
PWB	20.59 ± 4.51	0.267^**^	0.318^**^	0.562^**^	-0.333^**^
PD	10.90 ± 5.77	-0.283^**^	-0.256^**^	-0.430^**^	0.438^**^

Note: * indicates *p* < 0.05, ** indicates *p* < 0.01; # T1 = baseline measurement; T2 = follow-up measurement; FFMQ = Chinese version of the Five Facet Mindfulness Questionnaire-Short Form; IPAQ = International Physical Activity Questionnaire Short Form; PWB = Positive Well-Being of Subjective Exercise Experience Scale; PD = Psychology Distress of Subjective Exercise Experience Scale

#### Mediation analysis

From a longitudinal perspective, we further explored the relationship between trait mindfulness and physical activity, and confirmed whether exercise positive well-being and exercise psychological distress functioned as parallel mediators in the relationship between these two variables. In this section, we used trait mindfulness (T1) as the predictor variable, physical activity (T2) as the outcome variable, and included exercise positive well-being (T2) and exercise psychological distress (T2) as mediators in the parallel mediation model. Mediation analysis was conducted using the PROCESS 4.1 macro for SPSS.

**Fig. 2:**
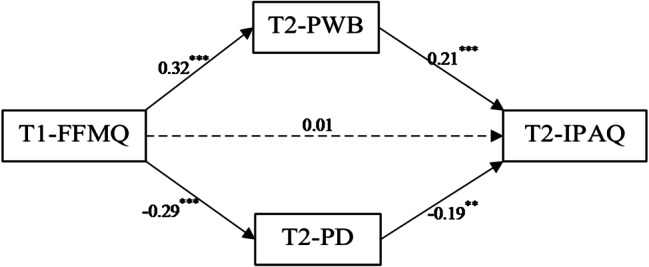
Mediating paths between trait mindfulness and physical activity. Note ** indicates *p* < 0.01, *** indicates *p* < 0.001; # T1 = baseline measurement; T2 = follow-up measurement; FFMQ = Chinese version of the Five Facet Mindfulness Questionnaire-Short Form; IPAQ = International Physical Activity Questionnaire Short Form; PWB = Positive Well-Being of Subjective Exercise Experience Scale; PD = Psychology Distress of Subjective Exercise Experience Scale

As shown in Fig. 2, trait mindfulness (T1) positively predicted exercise positive well-being (T2) (β = 0.32, *p* < 0.001) and negatively predicted exercise psychological distress (T2) (β = -0.29, *p* < 0.001). Exercise positive well-being (T2) positively predicted physical activity (T2) (β = 0.21, *p* < 0.001), while exercise psychological distress (T2) negatively predicted physical activity (T2) (β = -0.19, *p* < 0.01). However, trait mindfulness (T1) did not significantly predict physical activity (T2). This analysis indicates that exercise positive well-being (T2) and exercise psychological distress (T2) serve as parallel mediators in the relationship between trait mindfulness (T1) and physical activity (T2). While trait mindfulness (T1) did not directly predict physical activity (T2), its effect was mediated through the affective experiences related to exercise, supporting our hypothesis of parallel mediation.

Based on the mediation analysis, the results showed that the total effect was 10.130, with a 95% confidence interval of [2.848, 17.412]. The indirect total effect was 9.206, with a 95% confidence interval of [5.561, 13.740], accounting for 90.88% of the total effect. Specifically, within the indirect effects:

(1) The mediation effect for the pathway “trait mindfulness (T1) → exercise positive well-being (T2) → physical activity (T2)” was 5.177, with a 95% confidence interval of [2.201, 8.902], accounting for 51.11% of the total effect.

(2) The mediation effect for the pathway “trait mindfulness (T1) → exercise psychological distress (T2) → physical activity (T2)” was 4.029, with a 95% confidence interval of [1.611, 7.215], accounting for 39.77% of the total effect.

The direct effect was 0.924, with a 95% confidence interval of [-6.693, 8.541], accounting for 9.12% of the total effect. Since the confidence interval includes zero, the direct effect was not statistically significant. See Table 4 for details.

The results of the longitudinal follow-up survey support the findings from the cross-sectional survey, further validating our hypothesis that exercise positive well-being and exercise psychological distress play parallel mediating effect in the relationship between trait mindfulness and physical activity.

**Table 4:** Results of mediation analyses (*n* = 354)

			95% CI		
Effect Type	Estimate	S.E.	Lower 2.5%	Upper 2.5%	|IE/DE|
Total Effect	10.130	3.703	2.848	17.412	
Indirect Total Effect	9.206	2.128	5.561	13.740	90.88%
FFMQ(T1) →PWB(T2) →PA(T2)	5.177	1.695	2.201	8.902	51.11%
FFMQ(T1) →PD(T2) →PA(T2)	4.029	1.437	1.611	7.215	39.77%
Direct Effect	0.924	3.873	-6.693	8.541	9.12%

Note: S E = standard error, CI = confidence interval; IE = indirect effect (each path); DE = direct effect. FFMQ = Chinese version of the Five Facet Mindfulness Questionnaire-Short Form; IPAQ = International Physical Activity Questionnaire Short Form; PWB = Positive Well-Being of Subjective Exercise Experience Scale; PD = Psychology Distress of Subjective Exercise Experience Scale. T1 = baseline measurement; T2 = follow-up measurement

## Discussion

This study explored the relationship between trait mindfulness and physical activity, as well as the potential mediating role of exercise affective experiences, through a combination of cross-sectional and longitudinal surveys. The cross-sectional survey, utilizing a large sample size, included online self-report data from 853 participants. The results of the data analysis generally supported our hypothesis: exercise positive affective experiences and exercise negative affective experiences played parallel mediating effect in the relationship between trait mindfulness and physical activity. Building on this, we conducted a longitudinal follow-up survey, collecting self-reported assessments from 354 participants of the cross-sectional survey three months later. This allowed us to verify our findings from a cross-temporal perspective and deepen our understanding of the stable relationships among the study variables as they evolve over time.

Based on the results of the correlation analysis, our two surveys found significant correlations between trait mindfulness, exercise positive well-being, exercise psychological distress, and physical activity, which is consistent with recent research. Liu et al. (2023) found that trait mindfulness has a positive effect on physical activity [34]. Furthermore, affective mechanisms have been repeatedly highlighted and are considered key to more effectively understanding and predicting the initiation and maintenance of physical activity [51, 52]. However, we did not observe a significant correlation between physical activity (T1) and trait mindfulness (T2) in the longitudinal follow-up survey. This, in part, suggests that other intervening factors, such as seasonal and temperature changes, may have influenced the relationship between the two, potentially hindering participation in physical activity.

In the mediation analysis, our study primarily supports the parallel mediation effect of exercise positive well-being and exercise psychological distress in the relationship between trait mindfulness and physical activity. Specifically, trait mindfulness positively predicts exercise positive well-being and negatively predicts exercise psychological distress, while exercise positive well-being positively predicts physical activity, and exercise psychological distress negatively predicts physical activity. This result has been partially supported by previous research. Blanke et al. (2018) emphasized the different values of the two core characteristics of mindfulness on affective experiences, suggesting that being present in the moment helps increase positive affect, while maintaining a non-judgmental attitude towards current experiences helps reduce negative affect [29]. At the same time, affective experiences, as a motivational variable, play a crucial role in the activation and maintenance of health behaviors, including physical activity [38]. The Affect and Health Behavior Framework proposed outlines the impact of affect on physical activity. They argue that the affective responses to previous exercise behaviors can influence future exercise behaviors through the pathway of automatic affective processing. For instance, post-exercise release and satisfaction can more easily trigger subsequent exercise behaviors. Both our cross-sectional survey and longitudinal follow-up survey reveal that trait mindfulness does not appear to directly predict physical activity, which aligns with previous research [26, 53]. Tsafou et al. (2017) show that trait mindfulness is related to physical activity via an indirect path, namely through two consecutive mediators: first, state mindfulness, and then satisfaction [26].

Overall, this may suggest that trait mindfulness is associated with increased exercise positive affective experiences and decreased exercise negative affective experiences, which in turn helps promote physical activity. A previous study examined the impact and mechanisms of trait mindfulness on various health behaviors through a mediation model, showing that greater mindfulness may have downstream stress-reductive effects that enhance engagement in healthy behaviors, including physical activity [54]. Kang et al. (2017) found that higher levels of mindfulness could predict higher exercise motivation, with this effect mediated by negative affect [55]. Unfortunately, this study did not examine the role of positive affect. Zhang et al. (2023) supplemented this with their cross-sectional study, finding that eudaimonic well-being might mediate the connection between mindfulness and physical activity [50]. These studies all highlight the role of affect in the relationship between mindfulness or trait mindfulness and physical activity. Importantly, we examined the potential affective mechanisms between trait mindfulness and physical activity across different time frames. The cross-sectional survey enabled the rapid collection of data from a large sample at a specific point in time, providing preliminary insights into the relationships between variables, while the longitudinal follow-up survey revealed how these relationships evolve over time, offering more reliable connections.

It is clear that trait mindfulness has potential benefits in promoting physical activity, and existing research suggests that mindfulness training can enhance both mindfulness levels and trait mindfulness [56, 57]. This provides an effective strategy for promoting physical activity interventions: mindfulness training. For example, the body scan in the traditional mindfulness intervention MBSR encourages participants to gradually focus their attention on different parts of the body, thereby increasing awareness of their current physical state. This process helps reduce stress and improve mindfulness levels [25]. Mindfulness practices are also integrated into everyday physical activities. For example, focusing on the water temperature and sensation while washing dishes, or paying attention to the feeling of each step while walking [58].

In the context of promoting physical activity, enhancing positive affect, as a form of affective regulation, should also be trained. Exercise beginners can try using mindfulness techniques to focus on positive physical sensations during the activity, reducing attention to negative sensations and fatigue. If discomfort, pain, or fatigue arise during exercise, appropriately adjusting exercise goals and engaging in positive self-talk can help manage these negative affective experiences [59, 60], making it easier to experience positive emotional states during exercise.

It is worth mentioning that although our study was conducted in the second half of the year, influenced by seasonal changes and temperature drops, and although participants showed a noticeable decline in physical activity levels at T2 compared to T1 in the longitudinal follow-up, our cross-time mediating analysis results revealed that exercise positive well-being and exercise psychological distress maintained their mediating effect between trait mindfulness and physical activity. Similarly, a study by D MEYER et al. (2018) found that mindfulness training could mitigate the impact of shorter daylight and cooler weather on physical activity participation [61]. These findings suggest that trait mindfulness may help alleviate the negative effects of seasonal changes on physical activity. This insight encourages us to consider incorporating mindfulness training into physical activity promotion programs in the future to help relevant groups better adapt to environmental changes. Additionally, our findings provide evidence in response to a recent study by Jose & Geiserman (2024), which found that trait mindfulness effectively predicted a reduction in negative affective experiences over three months but did not predict an increase in positive affective experiences [33]. Similarly, the longitudinal follow-up results of our study indicated that trait mindfulness effectively predicted a reduction in exercise negative affective experiences and additionally revealed an increase in exercise positive affective experiences. This supports our expectations regarding the relationship between trait mindfulness and exercise affective experiences, and suggests a possible interaction between physical activity and positive affective experiences.

Overall, this study has several notable strengths. First, our findings identified the parallel mediation effects of exercise positive well-being and exercise psychological distress between trait mindfulness and physical activity, providing new empirical support for explaining the potential mechanisms through which trait mindfulness promotes physical activity. Secondly, our study found that mindfulness-based physical activity intervention strategies appear to be a more effective and convenient option, providing a new approach for the development of future physical activity intervention strategies. Finally, by combining cross-sectional and longitudinal studies, this research explored the relationship between trait mindfulness and physical activity, revealing the variation and stability of these variables across different time stages.

Of course, there are some limitations. First, our study does not directly verify the effectiveness of mindfulness training in promoting physical activity. Although we conducted cross-sectional and longitudinal investigations, more randomized controlled trials are needed in the future to validate the efficacy of this mindfulness-based intervention strategy. Secondly, our research population consists of ordinary college students, and the sample is relatively homogeneous. Their average physical activity level is slightly lower than that of similar samples in other studies [62], which may influence the external validity of the research findings. The promotion of physical activity may be more valuable for sedentary individuals, those with chronic diseases, and the elderly. Future research could focus on these specific groups to explore meaningful interventions. Third, this study relied on online self-reported measures of physical activity, which may not fully capture objective data. To enhance accuracy, future studies could consider using accelerometers or other objective devices to measure physical activity levels. Finally, physical activity is a long-term process, and unfortunately, the longitudinal follow-up in this study lasted only three months. Future research could extend the time frame to explore the effects of trait mindfulness and mindfulness training on promoting physical activity, especially in terms of their role in maintaining physical activity amidst various environmental or life changes.

## Conclusions

This study explored the relationships among trait mindfulness, exercise positive well-being, exercise psychological distress, and physical activity through a combination of cross-sectional and longitudinal designs. The results revealed that trait mindfulness positively predicted exercise positive well-being and negatively predicted exercise psychological distress. Exercise positive well-being was found to positively predict physical activity, while exercise psychological distress negatively predicted physical activity. Additionally, exercise positive well-being and exercise psychological distress both played parallel mediating effect in the relationship between trait mindfulness and physical activity.

## Data Availability

Data is provided within the manuscript or supplementary information files.

## Abbreviations

MBSR: Mindfulness-Based Stress Reduction
DMT: The Dual Mode Theory
ART: The Affective Reflective Theory
AHBF: The Affect and Health Behavior Framework
BMI: The Body Mass Index
FFMQ-C-SF: The Chinese Short Form of the Five Facet Mindfulness Questionnaire
SEES: The Subjective Exercise Experience Scale
PWB: Positive Well-Being
PD: Psychological Distress
IPAQ-SF: The International Physical Activity Questionnaire Short Form
